# Identifying functional and regional differences in chimpanzee stone tool technology

**DOI:** 10.1098/rsos.220826

**Published:** 2022-09-21

**Authors:** Tomos Proffitt, Jonathan. S. Reeves, Soiret Serge Pacome, Lydia. V. Luncz

**Affiliations:** ^1^ Technological Primates Research Group, Max Planck Institute for Evolutionary Anthropology, Deutscher Platz 6, 04103 Leipzig, Germany; ^2^ Laboratoire de Zoologie et de Biologie Animale, Université Félix Houphouët-Boigny, Abidjan, 22 BP 582 Abidjan 22, Côte d'Ivoire, West Africa

**Keywords:** primate archaeology, nut-cracking, stone tools, percussive technology, Palaeolithic, archaeology

## Abstract

The earliest hominin archaeological sites preserve a record of stone tools used for cutting and pounding. Traditionally, sharp-edged flakes were seen as the primary means by which our earliest ancestors interacted with the world. The importance of pounding tools is increasingly apparent. In some cases, they have been compared with stone hammers and anvils used by chimpanzees for nut-cracking. However, there has been little focus on providing a robust descriptive and quantitative characterization of chimpanzee stone tools, allowing for meaningful comparisons between chimpanzee groups and with archaeological artefacts. Here we apply a primate archaeological approach to characterize the range of chimpanzee nut-cracking stone tools from Djouroutou in the Taï National Park. By combining a techno-typological analysis, and two- and three-dimensional measures of damage, we identify clear differences in the location and extent of damage between nut-cracking hammerstones and anvils used at Djouroutou and when compared with other wild chimpanzee populations. Furthermore, we discuss these results in relation to interpretations of Plio-Pleistocene percussive technology. We highlight potential difficulties in identifying the underlying function of percussive artefacts based on morphological or techno-typological attributes alone. The material record from Djouroutou represents an important new datum of chimpanzee regional and material culture.

## 1. Introduction

Our earliest ancestors used a variety of stone tools to interact with, and modify the world around them [1–4]. Tools used for cutting and pounding tasks provided a competitive advantage in accessing different food sources, thus influencing the cultural and biological evolution of our species [5,6]. The role of percussive artefacts for understanding Plio-Pleistocene hominin subsistence has received far less attention compared with stone flake technology [7–10]. Both hammerstones and anvils used for percussive behaviours have been identified at several Plio-Pleistocene archaeological sites and indeed form a component of archaeological assemblages into the Later Stone Age [1,7,9–14].

The techno-typological characteristics of tools used for percussive activities are heavily dictated by the underlying raw material properties, morphologies, the degree to which they were used and the materials on which they were used. Archaeological percussive artefacts can be grouped into active and passive elements [15]. Active elements comprise typical handheld hammerstones [7], while passive elements consist of stationary anvils on which various materials were pounded. Both anvils and hammerstones are identified in the archaeological record by a combination of morphology and percussive damage patterns on one or more active (used) surfaces. These include pitting, crushing, and the development of depressions, striations and the detachments of unintentional flakes.

Traditionally, hammerstones in the archaeological record included only those used for knapping activities characterized as rounded cobbles with intensive battering and pitting on convex cortical surfaces [1]. It is apparent that a greater diversity of hammerstone types exists in the Plio-Pleistocene archaeological record, including hammerstones with fracture angles [7], subspheroids and spheroids [7,16]. These may have been used for activities such as bone-breaking [7] or represent cores that have been re-used as hammerstones [16,17].

Anvils appear in the Plio-Pleistocene record as early as 3.3 Ma [18] at Lomekwi 3 in West Turkana (Kenya). These are interpreted as having multiple possible functions. First as anvils for bipolar flaking or other percussive tasks, and secondarily as passive hammers, where handheld cores were struck against them to detach large flakes [18,19]. During the Oldowan (2.6–1.6 Ma), anvils are found in low frequencies throughout East Africa, including Olduvai Gorge [1,7,9,20], Melka Kunturé [15,21] and West Turkana [10]. Both active and passive stone tools are also found at Acheulean (1.6–0.8 Ma) sites in East Africa including Olduvai Gorge [20], Melka Kunturé [21] and in the Levant, including Gesher Benot Ya'aqov [13,22], Ubeidiya [23] and Latamne [24].

Artefacts interpreted as anvils often possess a cuboid [1] or trapezoid [22] morphology with edges that are roughly 90^o^, a flat active surface with evidence of percussive damage, a flat base which would have provided stable support and occasionally possess flake scars along their edges [1,22]. At Olduvai, tabular quartzite blocks with two flat surfaces are identified as anvils [20] as well as some smaller pitted igneous cobbles [1,11]. Suggested uses for these artefacts include bipolar flaking [25,26], bone-breaking [27], flake-splitting [17] or potentially other percussive behaviours such as nut-cracking [25]. At Gesher Benot Ya'aqov identified anvils have been associated with nut-cracking [13]. These are often ingenious tabular blocks which possess one active surface containing one or more pits [22]. Additionally, in some cases, flake detachments around the periphery have been interpreted as a stage of intentional anvil shaping [22].

In recent years, researchers have sought to better understand the formation and function of hominin percussive tools by drawing on modern replicative experimental studies [25,26,28], techno-typological as well as functional and use-wear analyses of artefacts [7,9,10,29,30]. Primate archaeological methods have also developed as a valid means by which to better understand percussive technology [31,32]. Primate archaeology seeks to link the observed dynamic tool use behaviours of various modern primates to their resulting static archaeological record. This is achieved by describing the variation within and between primate stone tool uses and their resulting assemblages [31]. In doing so, an expanded referential framework for archaeological artefacts can be developed. Recently, there have been strides towards increasingly detailed documentation of percussive tools used by both captive [33] and wild primates, including capuchin monkeys (*Sapajus libidinosus*) [34–36], long-tailed macaques (*Macaca fascicularis*) [37,38] and chimpanzees (*Pan troglodytes verus*) [39,40].

Due to the close phylogenetic relationship with humans, chimpanzees have often been used as a comparative model for investigating a range of hominin behaviours, including the emergence of tool use [41–43], raw material selection [44,45], site formation [44,46], learning and cognition [47–49], tool transport patterns [46,50,51] and for understanding the range of forms and functions of different hominin stone tools [13,18,33,52]. Chimpanzee stone tool use is known from a number of locations in West Africa [53]; however, long-term detailed documentation of stone tool use among wild chimpanzees is currently only known from two regions. The first, the forest of Bossou in Guinea, is home to a small group of chimpanzees who use both stone hammers and anvils to crack oil palm nuts (*Elaeis guinneensis*) [54–56]. It has been suggested that this group of chimpanzees represents a useful comparative model for early hominin tool use, as their hammerstones and anvils are relatively small and both are readily transported [39,42,44,57]. The second are the chimpanzees who inhabit the Taï National Park in Côte d'Ivoire [58,59]. The chimpanzees of the Taï forest are known to use both stone and wooden tools to crack a wide variety of nut species (these include *Coula edulis, Panda oleosa, Parinari excelsa, Sacoglottis gabonensis* and *Detarium senegalense*) [58,60,61]. To date, the majority of published information regarding chimpanzee tool use in the Taï forest has concentrated on four groups from the Taï Chimpanzee Project, close to the village of Taï [50,51,62–64].

Wild chimpanzee hammerstones have been directly compared with archaeological lithic material and assemblages [44,52,65]. Most of these comparisons have focused on aspects such as density of material [44], mean dimensions and mass of hammerstones compared with hominin hammerstones [18,52,65] and broad typological descriptions of hammerstones as pitted objects [22,66]. Recently, more quantitative analyses of chimpanzee stone tools have focused on two- and three-dimensional geographic information system (GIS) characterization of a sample of tabular hammerstones and anvils used by chimpanzees from Bossou (Guinea) to crack oil palm nuts [39]. Here, various surface morphometric measures, as well as the spatial mapping of damaged areas, were used to quantify damage patterns.

The only other recent systematic analysis of chimpanzee stone percussive material from outside Bossou was conducted by Proffitt *et al.* [40] on the lithic assemblage from the Panda 100 site in the Taï Forest [52]. This study provided an updated techno-typological and microscopic use-wear characterization of a large fragmented assemblage excavated from multiple wooden nut-cracking anvils used by chimpanzees. This assemblage was represented by a high frequency of angular fragments due to the friability of the predominant raw material used as hammerstones. A small frequency of these artefacts preserved microscopic crushing and scarring typical of percussion which may be identifiable in archaeological contexts. This assemblage contained no artefacts which could be considered functional hammerstones, diminishing its efficacy as a comparative dataset for hominin percussive tools [40].

To develop a better understanding of the variation within and between chimpanzee stone tools used for nut-cracking, a comprehensive referential database that includes different populations, raw materials used and nut species cracked is essential. In this study, we document and characterize the nut-cracking stone tools, both hammerstones and anvils, used by chimpanzees within the study area of Djouroutou in the South of the Taï National Park. The chimpanzees of Djouroutou use stone hammers and stone and wooden anvils to crack a wide variety of nut species [60,61]. By combining a techno-typological analysis, along with two- and three-dimensional quantifiable measures of percussive damage patterns, we address the following research questions (i) what are the differences between chimpanzee nut-cracking hammerstones and anvils at Djouroutou and (ii) are there differences between the hammerstones and anvils used at Djouroutou and other chimpanzee and hominin percussive tools? The second research question is addressed by directly comparing our data with published data of percussive tools used by chimpanzees from Bossou [39] and by discussing the results of this study in relation to other published primate and hominin percussive tool descriptions. By doing so, this study expands the referential comparative dataset by which hominin percussive artefacts can be compared and interpreted. In a primate archaeological sense, the lithic record from Djouroutou represents a new datum point for chimpanzee regional and material cultural comparison of stone tool morphology and use [61].

## 2. Material and methods

### 2.1. Materials

#### 2.1.1. Study site

**Figure 1.**
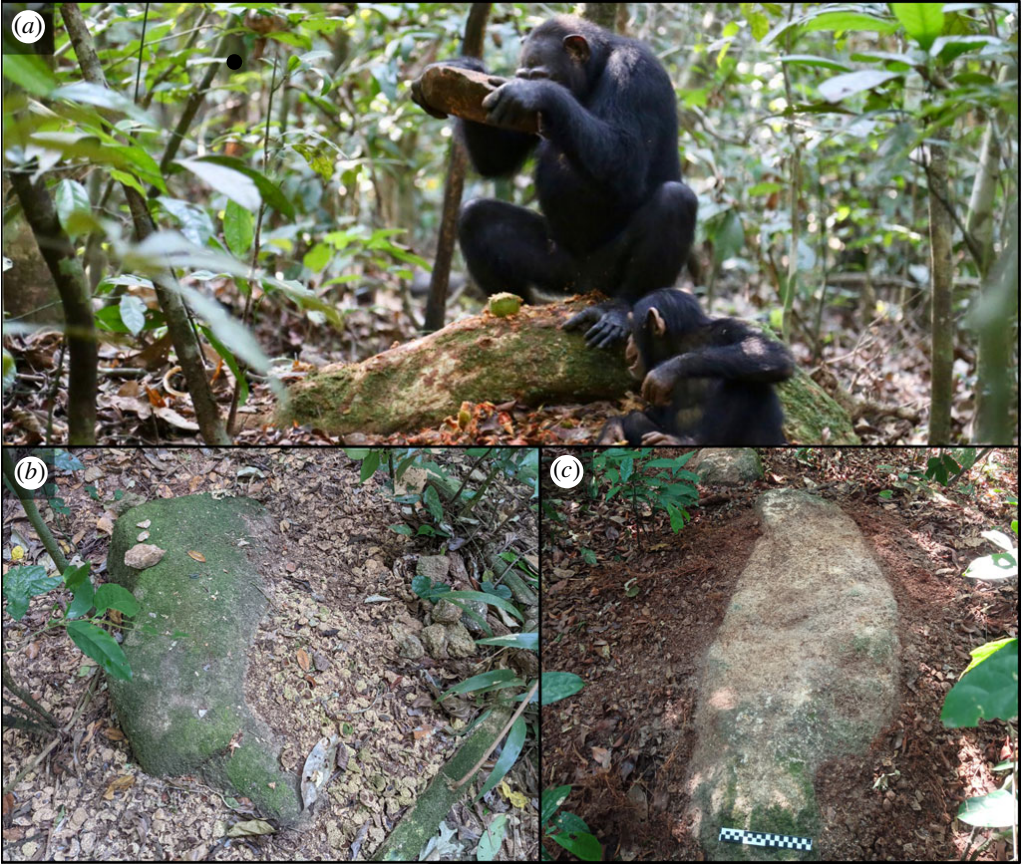
Examples of chimpanzee nut-cracking in the Taï forest and a nut-cracking site at Djouroutou. (*a*) Female chimpanzee cracking *Panda oleosa* nuts using a granodiorite hammerstone on a wooden (panda tree root) anvil (Credit: Liran Samuni, Taï Chimpanzee Project), and (*b,c*) examples of an active *Panda oleosa* nut-cracking site at Djouroutou. Note the combination of hammerstone, anvil and fresh nut debris.

The chimpanzee field site of Djouroutou is located in the southwest of the Taï National Park in Côte d'Ivoire and situated 12 km from a village of the same name. This area is home to a group of around 60 wild chimpanzees, who occupy a territory of roughly 25 km^2^ [60] framed in the north by the Hana river. The chimpanzees of Djouroutou regularly use stone and wooden tools to crack open five species of nut [60]. These include coula (*Coula edulis*), panda (*Panda oleosa*), parinari (*Parinari excelsa*) saccaglottis (*Sacoglottis gabonensis*) and more rarely detarium (*Detarium senegalense*) [60]. Compared with the research site of the Taï Chimpanzee Project further north, the Djouroutou chimpanzees only use stone hammers. These are used in conjunction with both wooden and stone anvils [61]. Wooden anvils generally take the form of living tree roots, often, on the nut tree itself [60], while stone anvils are often, but not exclusively, semi-exposed boulders (figure 1*a–c*). A range of raw materials are used as both hammers and anvils at Djouroutou; these include quartzite, granodiorite, metamorphosed granite, and to lesser extent laterite [60].

#### 2.1.2. Hammerstone and anvil sample

We selected a total of 11 hammerstones and seven stone anvils for this study. Each hammerstone and anvil was selected based on a contextual criterion to ensure that only actively used tools were included in the study. All hammerstones were found in close association with an active anvil (either wooden or stone) and freshly cracked nut debris. All anvils were found in association with an active hammerstone and freshly cracked nut debris in a radius of 50 cm to the anvil. The sample presented in this study represents the full range of the most common raw materials used as stone tools at Djouroutou, quartzite, granodiorite and metamorphosed granite. In all but one case, clear percussive damage is visible on all hammers and anvils. A single large quartzite hammerstone possesses no clear percussive damage. It is, however, included in our analysis for two reasons. First, its classification as a hammerstone is clear based on direct observations; it was found in direct association with an active wooden anvil and freshly cracked *Paranari excelsa* nut shells. Second, it represents a used hammerstone which possesses no apparent percussive damage and as such should be included in characterization of this tool type. The sample of hammerstones presented in this study was used to crack three of five species of nut eaten by the Djouroutou chimpanzees (coula, panda and parinari). Stone anvils presented in this study were used to crack panda, parinari and sacaglottis nuts.

### 2.2. Methods

To characterize and differentiate between the Djouroutou hammerstones and anvils, this study employs a combination of techno-typological and macro use-wear analyses. The techno-typological analysis presented here follows protocols set out by de la Torre *et al.* [25], used previously on both hominin [7,29] and primate [33] hammerstones and anvils. The analysis of macro use-wear presented here focuses on quantifying the spatial patterns and three-dimensional surface morphometry of discrete areas of pitting on both hammers and anvils. Discrete pits were chosen as a means of quantifying percussive damage as they are readily visible and can be clearly defined based on three-dimensional surface morphometric methods [67,68]. To assess similarities and differences in percussive damage between the tools from Djouroutou and those reported from Bossou, the results of this study were directly compared with published data on anvils [39], derived from the same spatial analytical methods presented in this study.

Data collection for this study was non-invasive and in compliance with the requirements and guidelines of the ‘Ministère de l'enseignement supérieure et de la recherche scientifique’, which granted permission for this research, and adhered to the legal requirements of the Côte d'Ivoire. All data collection also strictly adhered to the regulations of the Deutsche Tierschutzgesetz and the American Society of Primatologists' principles for the ethical treatment of non-human primates.

#### 2.2.1. Techno-typological analysis

Before analysis, all hammerstones and anvils were orientated following protocols set out by Goren-Inbar *et al.* [22]. Each artefact was rotated so that its maximum length ran parallel to a fixed *Y*-axis and the flat surface that possessed the greatest degree of visible percussive damage was faced upwards. Six idealized planes were then assigned to each artefact, Plane A representing the upward-facing active surface with Plane A2 representing the opposite face, Planes B and B2 are located on the forward and rear surfaces of the artefact and Plane C and C2 represent the left and right surfaces (electronic supplementary material, figure S1). Additionally, a qualitative assessment was made of the grain size for each raw material, with each specimen being grouped into fine-, medium-, coarse- or very-coarse-grained categories.

Technological attributes assessed for all hammerstones include the morphological shape of the hammerstone, a classification of the visible macro percussive damage present, the location of percussive damage, the number of active planes (surfaces that possess evidence of use) and the morphology of the active plane. A range of macro damage patterns was identified and documented. These include depressions, pitting, crushing, flake detachments and adhering residue. Depressions are defined here as areas of micro pitting (less than 5 mm) which are dispersed across the active surfaces. Pitting is defined as a larger (greater than 10 mm in maximum length) discrete depressed areas on an active surface. Pits were identified both visually and in combination with three-dimensional surface morphometric analysis (described below). Crushing is defined as either discrete or contiguous visible areas on planes where the stone grains possess a crushed morphology [69]. Percussive flake detachments were identified based on the presence of clear flake scars on one or more surfaces of the artefacts. Where flake scars were present, the number and maximum dimensions of any flake scars present on the hammerstones were documented as well as the location of the flaking surface and platform from which these flakes derived. Additionally, based on the location of flake scars on hammerstones, an assessment of the platform and dorsal cortex was made as well as the minimum number of potential dorsal scars associated with each flake removal.

#### 2.2.2. Two- and three-dimensional quantification of pits

A combination of two-dimensional GIS spatial analysis and three-dimensional surface morphometric approaches were used to quantify the macro pitting damage present on hammerstones and anvils. All analysis was conducted on scaled high-resolution three-dimensional models with photorealistic textures. Detailed three-dimensional models of all hammers and anvils were recorded in the field using photogrammetric techniques and processed in Agisoft Metashape (v. 1.6.5). A Nikon D850 with a 45.7-megapixel sensor was used to record each artefact resulting in models with a point density of 25 000 points per cm^2^.

Three-dimensional point cloud analysis can be a powerful tool for quantifying the surface morphometry of percussive tools [67,68,70]. To both identify and extract all discrete areas of pitting on the active surfaces of hammerstones and anvils, all three-dimensional models were imported into the open-source software CloudCompare [71] along with a corresponding convex hull computed in Meshlab [72]. Following protocols set out by Benito-Calvo *et al.* [67], the topographic position index using a neighbourhood radius of 20 mm (to ensure that pits were fully identified) was used to identify all depressions on each artefact. A pitted region was defined by the maximum contiguous extent of each depression and was extracted from the original point cloud. Three different surface morphometric attributes were then calculated for each discrete pit. These included the depth of each pit, calculated from the nearest point from the encompassing convex hull, the gradient of each pit, measured as the ratio change of the elevation over distance [71], and surface roughness, calculated with a neighbourhood window of 0.5 mm.

Subsequently, all pits were subjected to two-dimensional spatial quantification using QGIS [73] following a slightly modified methodology to that set out in de la Torre *et al.* [25]. Georeferenced and scaled orthomosaic plans of each active surface for hammerstones and anvils were processed in Agisoft Metashape and digital elevation models (DEMs) of each extracted discrete pit for all artefacts were computed in CloudCompare. Both orthomosaics and DEMs were imported into QGIS. Each active surface and pit DEM was converted into a scaled vector which was used to calculate several spatial attributes. The maximum dimensions (length and width) of each pit were calculated using the *x* and *y* measurements of an orientated bounding box. Total surface areas of both pits and active surfaces were recorded and used to calculate the relative area of each active surface affected by pitting (PA). Additionally, the density (D) of pitting on an active surface was estimated by dividing the total number of discrete pits by the total area of the active surface. Two additional measures were calculated to describe the position of pitting on the active surface. These included the minimum, maximum and mean distance from the geometric centre of pits to the centre of the active surface (DAC) and the distance of the centre of each pit to the nearest edge of the active surface (DAE).

#### 2.2.3. Statistical analysis

To identify possible differences in percussive damage between tool types, nut species, raw materials and between Djouroutou and Bossou percussive tools, a combination of descriptive statistics (minimum, maximum, mean and s.d.) and non-parametric statistical tests were employed.

To explore differences between the hammerstones and anvils from Djouroutou and Bossou, a principal component analysis was used. To test for differences in dimensions and damage patterns between two samples, we performed a Mann–Whitney U test, while significant differences between two or more samples were tested using a Kruskal–Wallis test. To determine the factors that drive these differences we used *post hoc* Dunn's test and accounted for multiple testing using a Bonferroni correction. Significant differences were determined using an *α* of 0.05. All statistical tests were calculated using Microsoft Excel and R (v. 6.3 [74]), and the principal component analysis was conducted using PAST (v. 3) [75].

## 3. Results

### 3.1. Techno-typological analysis

#### 3.1.1. Hammerstones

Three separate raw materials were used as hammers, granodiorite, quartzite and metamorphosed granite, and hammers were found in association with both wooden and stone anvils. A visual inspection and qualitative description of each raw material used for hammerstones indicate a range of grain sizes. Granodiorite possesses a comparatively medium grain size, while the quartzite ranges from medium to very coarse grain and the metamorphosed granite possesses a very coarse grain. Of the 11 hammerstones studied here, all but one possess macroscopic traces of percussive use. A single large quartzite hammerstone used for cracking parinari nuts on a wooden anvil possesses no identifiable traces of percussive damage and has been excluded from the description of damage below. As this artefact was used as a tool, it is considered in the discussion.

**Figure 2.**
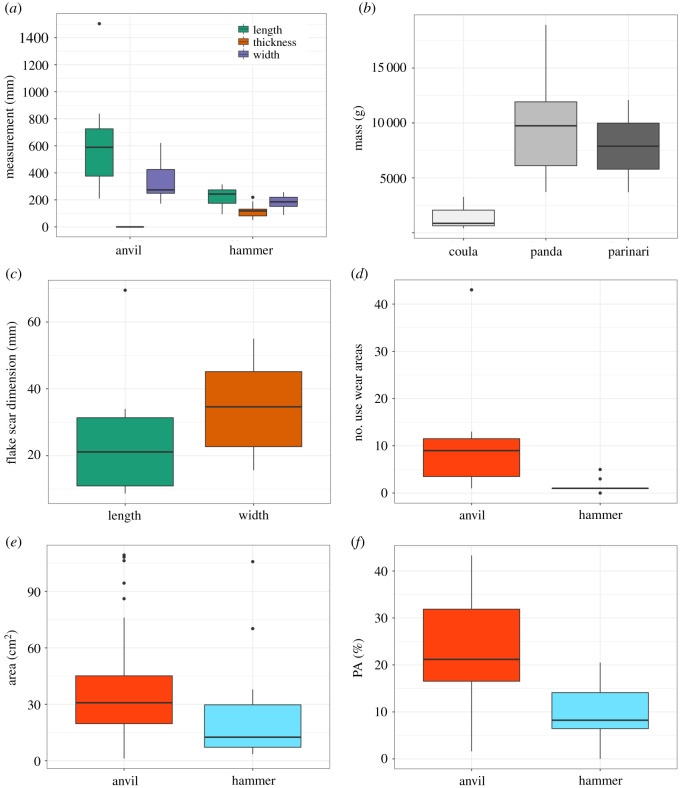
Descriptive statistics for all hammerstones and anvils are included in this study. (*a*) Maximum dimensions of hammerstones and anvils included in this study; (*b*) mass of all hammerstones separated by target nut cracked; (*c*) maximum technological length and width of all flake scars detached from hammerstones; (*d*) the number of discrete use-wear areas on both hammerstones and anvils; (*e*) the surface area (cm^2^) of all discrete use-wear areas on both hammerstones and anvils, and (*f*) the percentage of surface area (PA) occupied by discrete areas of percussive use-wear.

Hammerstones used by the chimpanzees at Djouroutou can be divided into two categories, pitted hammers (*n* = 7, 63.6%) and non-pitted hammers (*n* = 4, 36.4%). Hammerstones with clear pitting are generally associated with coarse- and very-coarse-grained raw materials (quartzite and metamorphosed granite), while non-pitted hammerstones are generally associated with medium- to fine-grained raw materials such as granodiorite and a single quartzite hammer. The hammerstones in this sample possess a mean length, width and thickness of 224.4 × 177.9 × 116.7 mm (figure 2*a*) and a mean volume of 2726 cm^3^ (±2208.26 cm^3^). There is no significant difference in length (Kruskal–Wallis, $\chi^{2}(2)=1.641$, *p* = 0.440), width (Kruskal–Wallis, $\chi^{2}(2)=1.664$, *p* = 0.435) nor thickness (Kruskal–Wallis, $\chi^{2}(2)=0.618$, *p* = 0.734) between the different raw materials used as hammerstones. There are significant differences in hammerstone volume (Kruskal–Wallis, $\chi^{2}(2)=6.060$, *p* = 0.048) and mass (Kruskal–Wallis, $\chi^{2}(2)=6.060$, *p* = 0.048) between nut species cracked (figure 2*b*). Hammerstones used to process coula nuts are notably smaller than those used to process both panda and parinari nuts, while there is little difference in dimensions between the latter two (figure 2*b*).

**Figure 3.**
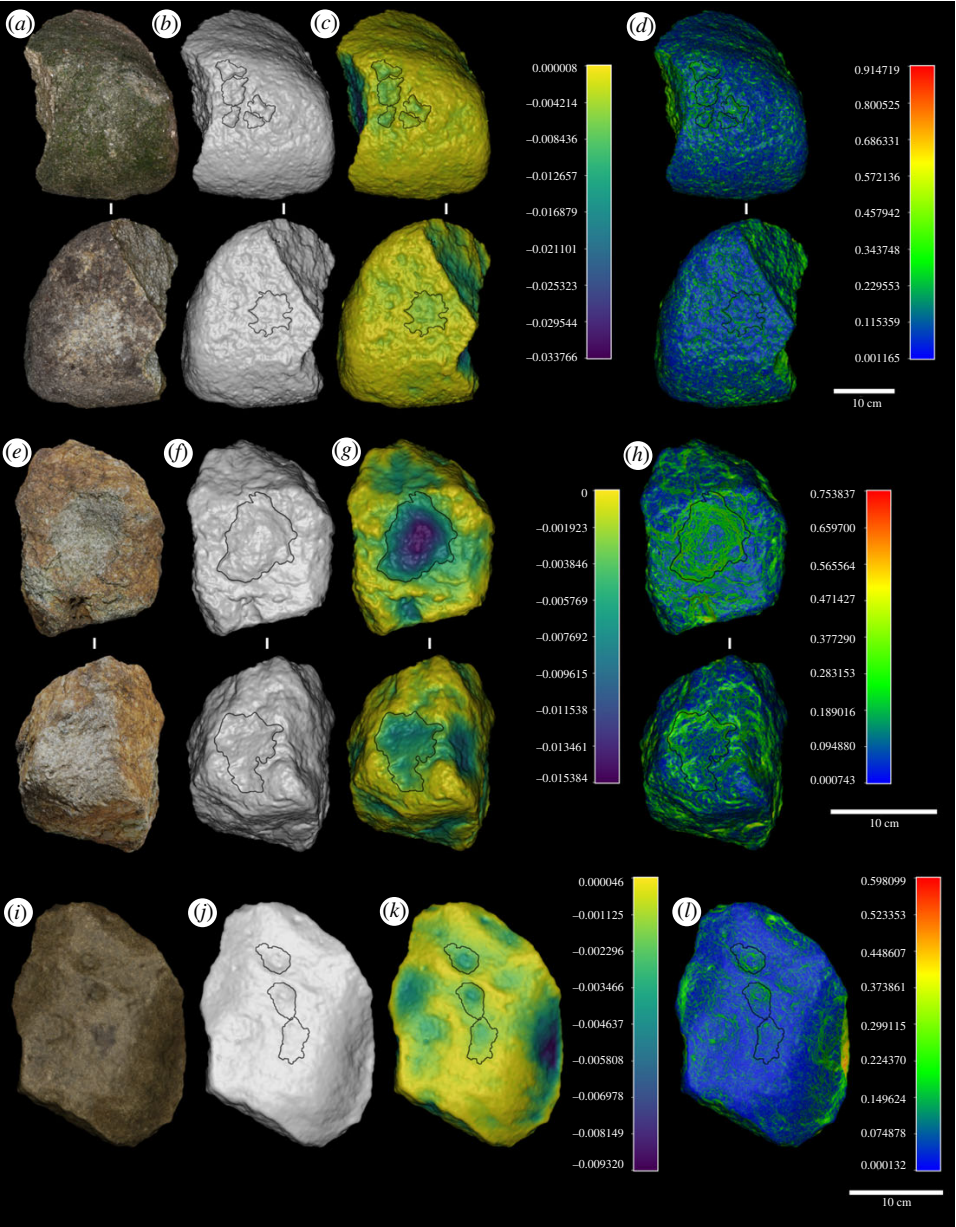
Examples of chimpanzee hammerstones (M Gra nite Hammer (*a–d*); PPQ1003 (*e–h*); CGG23 (*i–l*)) from Djouroutou included in this study illustrating their textured surface (*a*,*e*,*i*); three-dimensional surface (*b*,*f*,*j*); surface depth (mm) (*c*,*g*,*k*) and surface gradient (*d*,*h*,*l*) with location of all pits overlain.

Hammerstones generally possess a tabular morphology (*n* = 8, 72.7%); however, individual examples of plano-convex, trapezoidal and irregular morphologies are also present. Most hammerstones (*n* = 10, 90.9%) possess clear macroscopic evidence of use in the form of either percussive damage (*n* = 5, 45.5%), areas of residue (*n* = 1, 9%) or a combination of both (*n* = 4, 36.4%) (table 2). All hammerstones possess either one (*n* = 6, 54.5%) or two (*n* = 4, 36.4%) flat horizontal active surfaces, with damage generally located towards the centre of these planes (figure 3; electronic supplementary material, figures S2–S4).

A variety of different macro use-wear types are identified within the hammerstone sample. These include the formation of depressions, larger pitted regions, crushing, accidental flake detachments and adhering residue (table 2). Most hammerstones possess a combination of depressions and larger pitted regions (*n* = 7, 63.6%) often associated with visible crushing of the grains in and around the depressions, while four hammerstones possess clear flake detachments due to percussion. Depressions, pitting and crushing are more prevalent on coarse-grained quartzite and metamorphosed granite hammerstones, while flake detachments are only present on the medium-grained granodiorite hammerstones, which conversely possess fewer areas of pitting. In examples where pitting is present on the active surface, pits are generally circular in plan shape and possess a concave base. In the majority of cases where flake detachments are present, they occur in localized areas on and along vertical planes and originate from the horizontal flat active plane(s) of the hammerstones (electronic supplementary material, figure S5). However, on one hammerstone, the flake detachments originate from a vertical plane and are detached from an active plane. These percussive flake detachments are all non-invasive and remove very little volume; however, in some cases, flake scars are superimposed. In five cases, detached flakes would have preserved at least one unidirectional dorsal scar. All percussive flakes would have exhibited cortical platforms and 50% (*n* = 6) would have possessed non-cortical dorsal surfaces. The majority of the detached flakes would have possessed step terminations (*n* = 10, 83.3%) with two possessing feather terminations. Flake scars are generally short and wide, possessing mean technological lengths and widths of 24.7 × 33.9 mm but vary in dimensions considerably, with minimum and maximum lengths of between 8.7 and 69.5 mm and widths between 15.6 and 54.9 mm (figure 2*c*).

#### 3.1.2. Anvils

The stone anvils sampled in this study are large embedded blocks of metamorphosed granite (*n* = 6) or granodiorite (*n* = 1). These all have a relatively flat active surface which preserves clear evidence of percussive damage. Anvils tend to be large, possessing mean maximum lengths and width of 643.85 × 344.71 mm. These dimensions vary considerably with the length of some anvils ranging from 210 to 1504 mm (table 1). Overall anvils possess significantly larger active surfaces compared with hammerstones (Mann–Whitney U, U = 103, *p* = 0.0007) (figure 2*a*).

**Table 1.** General dimensions, raw material types and associated nut species of hammers and anvils presented in this study. Raw material abbreviations: Q = quartzite, GD = granodiorite, MG = metamorphose granite.

tool ID	tool type	raw material	nut species	max length (mm)	max breadth (mm)	max thickness (mm)	volume (cm^3^)	mass (g)
CGG23	hammer	GD	coula	264	189	61.8	1169.6	3275.0
CQQ34-18	hammer	Q	coula	136	87.8	71.9	329.2	855.8
M Granite Hammer	hammer	MG	panda	315	257	189	7282.4	18934.2
CTD1	hammer	Q	coula	93.1	90	50.7	158.8	412.9
PPGd	hammer	GD	panda	234	185	118	2771.3	7759.6
PGQ18	hammer	Q	panda	296	211	139	4513.4	11734.8
PPQ1003	hammer	Q	panda	185	140	123	1425.5	3706.3
PrGG1017	hammer	MG	parinari	164	163	90.4	1420.4	3693.1
PrPrQ 3015	hammer	Q	parinari	243	227	219	4646.9	12082.0
PWS5	hammer	GD	panda	284	172	100	1987.6	5565.3
PWS7	hammer	GD	panda	254	235	121	4281.1	11987.1
PGG3005-Ex	anvil	MG	panda	210	172	—	—	—
PGQ18-A	anvil	MG	panda	299	274	—	—	—
PGQ18-B	anvil	MG	panda	589	260	—	—	—
PGU3012	anvil	MG	panda	614	364	—	—	—
PGU3013	anvil	MG	panda	1504	621	—	—	—
PRGU3014	anvil	MG	parinari	838	485	—	—	—
SGdGd-A	anvil	GD	sacaglottis	453	237	—	—	—

**Figure 4.**
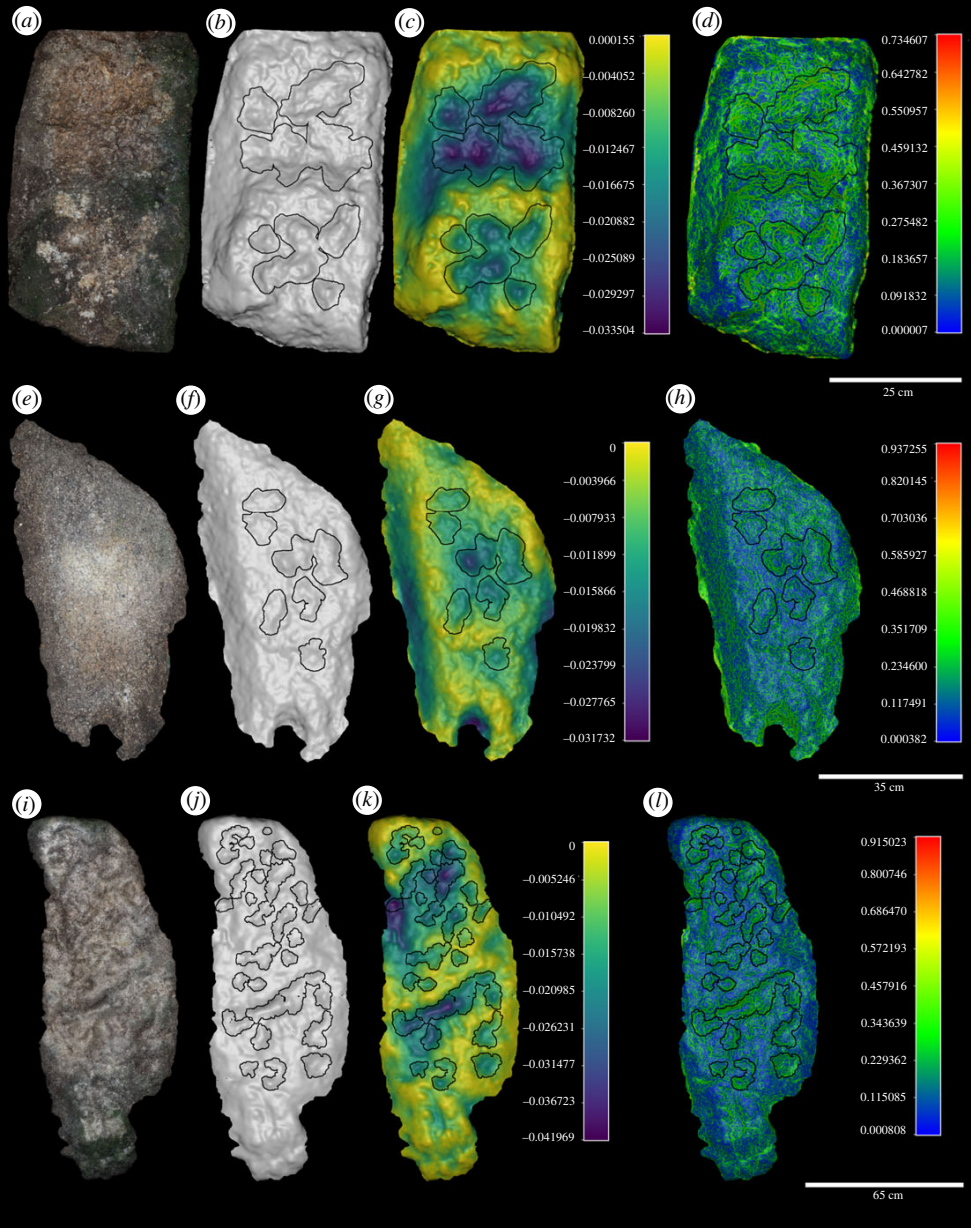
Examples of chimpanzee anvils (PGU3012 (*a–d*); PrGU3014 (*e–h*); PGU3013 (*i–l*)) from Djouroutou included in this study illustrating their textured surface (*a*,*e*,*i*); three-dimensional surface (*b*,*f*,*j*); surface depth (mm) (*c*,*g*,*k*) and surface gradient (*d*,*h*,*l*) with location of all pits overlain. All examples are metamorphosed granite.

All anvils possess small depressions and larger contiguous areas of pitting on their active surfaces, with the majority (*n* = 6) also possessing crushing in close association with pitted regions (table 2) (figure 4; electronic supplementary material, figure S6). However, differently to hammerstones, the anvils studied here do not possess any flake detachments nor do they show clear areas of residue adhesion. The lack of flake detachments can be associated with the coarse-grained nature of the raw material coupled with the lack of natural angles between platforms and potential flaking surfaces. The lack of clear macro-residue on and around pitted regions on anvils may be due to a higher degree of weathering associated with these stationary tools. The most prevalent form of macro damage present on all anvils are discrete areas of pitting distributed widely across the active surface. These mostly possess a circular plan shape, but in some cases, multiple closely located pits have combined to form either a singular elongated or irregularly shaped depression. In the majority of cases, pits possess an irregular base and steep edges.

**Table 2.** Overview of the macro wear present on all hammerstones and anvils included in this study.

tool ID	tool type	grain size category	macro wear	depressions	pitting	crushing	flake detachment	residue	no. discrete use-wear	no. active planes
CGG23	hammerstone	medium	yes	yes	yes	no	yes	yes	3	2
CQQ34-18	hammerstone	coarse	yes	yes	yes	yes	no	no	1	2
CTD1	hammerstone	coarse	yes	yes	yes	yes	no	yes	6	2
M Granite Hammer	hammerstone	very coarse	yes	yes	yes	yes	no	no	1	2
PPGd	hammerstone	medium	yes	no	no	no	yes	yes	1	2
PGQ18	hammerstone	coarse	yes	yes	yes	yes	no	yes	1	1
PPQ1003	hammerstone	coarse	yes	yes	yes	yes	no	no	2	2
PWS5	hammerstone	medium	yes	no	no	no	yes	yes	2	2
PWS7	hammerstone	medium	yes	yes	no	yes	yes	yes	0	2
PrGG1017	hammerstone	very coarse	yes	yes	yes	yes	no	no	2	2
PrPrQ_3015	hammerstone	fine	no	no	no	no	no	no	0	indeterminate
PGG3005-Ex	anvil	very coarse	yes	yes	yes	yes	no	no	1	1
PGQ18-A	anvil	very coarse	yes	yes	yes	yes	no	no	1	1
PGQ18-B	anvil	very coarse	yes	yes	yes	yes	no	no	13	1
PGU3012	anvil	very coarse	yes	yes	yes	yes	no	no	10	1
PGU3013	anvil	very coarse	yes	yes	yes	yes	no	no	43	1
PRGU3014	anvil	very coarse	yes	yes	yes	yes	no	no	9	1
SGdGd-A	anvil	medium	yes	yes	yes	no	no	no	5	1

Of note within the hammerstone sample presented here are two artefacts which bear a combination of percussive damage patterns which suggest their use as both hammerstones and anvils during their use life. The first is artefact CGG23, interpreted initially and collected as a hammerstone due to the presence of freshly cracked coula nuts, localized adhering organic residue and its deposition on a granite anvil. The second is a large fragmented metamorphosed granite hammerstone (M Granite Hammer) with percussive damage on two opposite planes. Damage on one plane is fresh, centrally located and consists of a singular sub-circular pit, while damage on the opposite plane consists of five separate smaller pits, distributed in a dispersed manner on the active surface and covered in algae growth (attesting to their older origin).

### 3.2. Two- and three-dimensional characterization of pits

Hammerstones possess on average 1.3 (±1.1) discrete pitted areas on each of their active surfaces. This is in stark contrast to the frequency of discrete pitted regions identified on anvils. On average, anvils possess 11.6 pits/depressions on their active surface; this ranges from a minimum of one to a maximum of 43 discrete pits (figure 1*d*). There is no significant difference in the density of discrete use-wear areas on hammerstones and anvils (Mann–Whitney U, U = 73, *p* = 0.277), nor is there a significant difference in density between raw materials for both hammerstones (Kruskal–Wallis, $\chi^{2}(2)=1.753$, *p* = 0.416), nor anvils (Kruskal–Wallis, $\chi^{2}(1)=0.25$, *p* = 0.617).

Discrete areas of pitting on hammerstones measure on average 55.73 (±24.8) × 49.12 (±28.3) mm, while pitted areas on anvils are slightly larger, with mean dimensions of 77.1 (±36.1) × 56.8 (21.6) mm (table 3). Pitted regions on anvils are significantly longer (Mann–Whitney U, U = 1214, *p* = 0.0056) than those on hammers, however, not significantly wider (Mann–Whitney U, U = 1087, *p* = 0.081), resulting in hammerstones possessing significantly smaller pitted areas (Mann–Whitney U, U = 1208, *p* = 0.0065) compared with anvils (figure 2*e*). There is no significant difference in pit surface area on hammerstones observed between raw material types (Kruskal–Wallis, $\chi^{2}(2)=0.504$, *p* = 0.777), nor between nut species cracked (Kruskal–Wallis, $\chi^{2}(2)=4.314$, *p* = 0.115). Metamorphosed granite hammerstones elicit the greatest maximum values and s.d. for the use-wear area (table 3) of each raw material. The differences in depression lengths between hammerstones and anvils may be due to the merging of multiple closely located individual depressions (discussed above).

**Table 3.** Spatial and three-dimensional morphometric characteristics of all discrete use-wear locations on chimpanzee hammerstones and anvils included in this study, separated by raw material types.

	hammerstones				anvils		
	GD	MG	Q	All	GD	MG	All
area (cm^2^)							
min	6.80	4.90	3.50	3.50	1.20	4.60	1.20
max	105.80	34.40	37.90	105.80	5.70	109.30	109.30
mean	33.66	17.09	17.73	22.80	2.46	37.31	35.21
s.d.	38.89	12.49	13.44	24.81	1.93	23.45	24.21
PA (%)							
min	3.81	6.10	0.00	0.00	1.63	14.24	1.63
max	20.50	15.89	19.09	20.50	1.63	43.29	43.29
mean	9.97	10.69	9.44	9.95	1.63	26.88	23.27
s.d.	6.09	4.83	7.89	6.17	—	10.88	13.78
DAC (mm)							
min	3.64	1.38	1.64	1.38	16.40	16.61	16.40
max	73.44	79.59	18.98	79.59	94.52	655.93	655.93
mean	20.86	28.55	10.27	20.76	51.92	246.81	235.07
s.d.	24.91	27.24	7.45	22.74	31.63	177.87	178.70
DAE (mm)							
min	3.64	1.38	1.64	7.22	16.40	16.61	33.36
max	73.44	79.59	18.98	105.90	94.52	655.93	213.99
mean	20.86	28.55	10.27	68.83	51.92	246.81	111.09
s.d.	24.91	27.24	7.45	24.17	31.63	177.87	46.01
UW density							
min	0.0019	0.0020	0.0000	0.0000	0.0066	0.0033	0.0033
max	0.0081	0.0085	0.0167	0.0167	0.0066	0.0106	0.0106
mean	0.0036	0.0049	0.0068	0.0051	0.0066	0.0056	0.0057
s.d.	0.0023	0.0027	0.0063	0.0043	—	0.0027	0.0025
depth (mm)							
min	−11.2850	−13.9360	−15.3590	−15.3590	−15.2090	−41.9490	−41.9490
max	0.0090	−1.7400	−1.8940	0.0090	−4.6910	0.3070	0.3070
mean	−2.5492	−5.8334	−6.8202	−4.3116	−10.0060	−15.5713	−15.4852
s.d.	2.3305	2.3753	3.1235	3.1230	2.2548	7.4075	7.3873
gradient							
min	0.0003	0.0017	0.0013	0.0003	0.0013	0.0007	0.0007
max	0.4387	0.6378	0.5589	0.6378	0.4680	0.6523	0.6523
mean	0.0702	0.1681	0.1781	0.1191	0.1406	0.1871	0.1863
s.d.	0.0432	0.0878	0.0825	0.0843	0.0713	0.0940	0.0939
roughness (mm)							
min	0.0000	0.0000	0.0000	0.0000	0.0000	0.0000	0.0000
max	0.8700	1.3550	1.3130	1.3550	1.1920	2.7890	2.7890
mean	0.1111	0.2430	0.2199	0.1703	0.2088	0.2481	0.2475
s.d.	0.0921	0.1940	0.1732	0.1579	0.1758	0.2076	0.2072

Hammerstones possess significantly less damage on their active surfaces than anvils (Mann–Whitney U, U = 92, *p* = 0.0149), with a mean PA value of 9.95% (±6.2%) and maximum value of 20.4%. Anvils on the other hand possess a mean PA value of 23.3% (±13.7%) with a maximum of 43.3%. For hammerstones alone, there is no significant difference in PA values between raw materials nor between nut species cracked. Percussive damageon hammerstones is primarily located centrally on the active surface. This is corroborated by significantly lower DAC (mean = 20.7 (±22.7) mm) values on hammerstone active planes compared with anvils (mean = 235 (±178.7) mm) (table 3).

**Figure 5.**
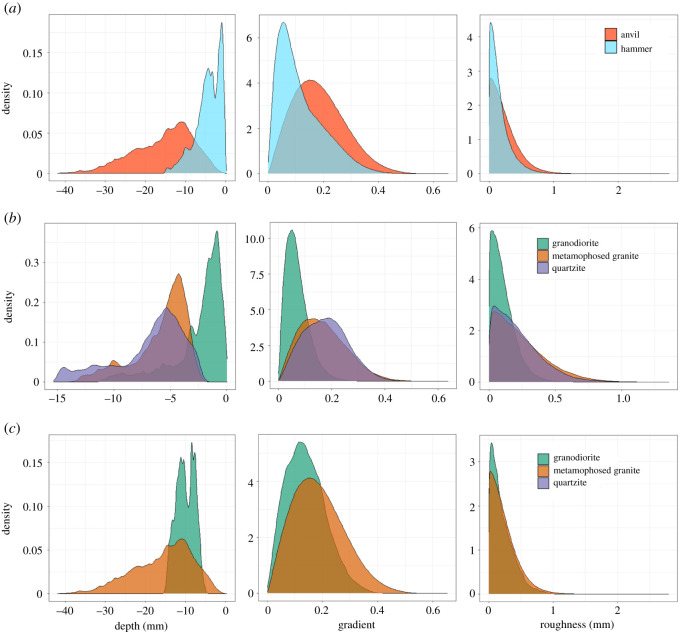
Density plot of depth, gradient and surface roughness values for all use-wear regions between (*a*) hammerstones and anvils, (*b*) different raw materials on hammerstones alone and (*c*) raw materials on anvils alone.

Pitted areas on hammerstones are significantly more shallow compared with those on anvils (Mann–Whitney U, U = 30, *p* = <0.001) (figure 5*a*). Hammerstone pits possess a mean depth of 4.31 (±3.12) mm, while those on anvils possess a mean depth of 15.48 (±7.38) mm. These differences are maintained across raw material types; however, pitted regions on hammers of the finer-grained granodiorite are significantly shallower compared with more coarse-grained raw materials such as quartzite (Mann–Whitney U, *p* = 0.033) and metamorphosed granite (Mann–Whitney U, *p* = 0.018) (figure 5*b*). No significant difference (Kruskal–Wallis, $\chi^{2}(1)=3.03$, *p* = 0.082) is noted for mean depth values of pitted regions between granodiorite and metamorphosed granite anvils. However, pits on metamorphosed granite anvils possess a far greater maximum depth (table 3; figure 5*c*).

The same pattern is observed when considering the mean gradient of pitted regions on both hammers and anvils. Hammerstones possess significantly lower mean gradient values compared with anvils (Mann–Whitney U, U = 1216, *p* = 0.005) (figure 2*a*), with a mean value of 0.119 compared with 0.186 for anvils (table 3). A significant difference between raw materials is also present (Kruskal–Wallis, $\chi^{2}(2)=10.837$, *p* = 0.004), with granodiorite hammers possessing significantly lower mean gradient values compared with metamorphosed granite (Mann–Whitney U, *p* = <0.001) and quartzite (Mann–Whitney U, *p* = 0.033) (figure 5*b*). The same pattern is observed for anvils (Kruskal–Wallis, χ^2^(1) = 13.223, *p* = <0.001), with granodiorite possessing significantly less steep pitted regions compared with metamorphosed granite (Mann–Whitney U, *p* = <0.001) (figure 5*c*).

Conversely, when considering the surface roughness of pitted regions on both hammerstones and anvils, no significant difference is identified (Mann–Whitney U, U = 1089, *p* = 0.078) (figure 4*a*). A significant difference is identified in roughness values between all raw material types for both hammerstones (Kruskal–Wallis, $\chi^{2}(2)=12.095$, *p* = 0.002) and anvils (Kruskal–Wallis, $\chi^{2}(1)=6.875$, *p* = 0.008). For both hammer and anvils, the harder and finer-grained granodiorite results in significantly smoother pitted regions compared with metamorphosed granite (Mann–Whitney U, hammerstones: *p* = <0.001; anvil: *p* = 0.009).

Raw material plays a substantial part in the formation of percussive damage patterns on both hammerstones and anvils. When raw material remains the same, anvils tend to possess deeper depressions associated with greater gradients in both granodiorite and metamorphosed granite. However, roughness levels within each of these raw materials show no significant variation between hammerstones or anvils.

### 3.3. Comparison with hammerstones and anvils from Bossou

**Figure 6.**
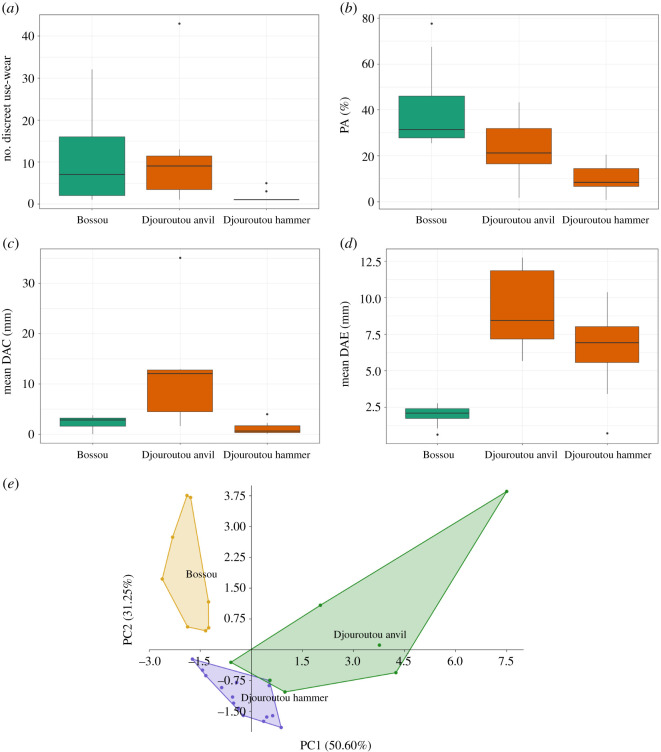
Comparative analysis between chimpanzee hammerstones and anvils from Djouroutou and anvils reported from Bossou [39]. Boxplots showing differences in (*a*) the number of discrete use-wear areas, (*b*) the relative surface area affected by percussive damage (PA), (*c*) the mean distance from the centre and (*d*) edge of the active surface to the geometric centre of discrete use-wear areas and (*e*) the results of a principal component analysis showing differences between the anvils and hammers of Djouroutou and with the anvils from Bossou using all measures listed in table 4.

Hammerstones and anvils from Djouroutou, Côte d́Ivoire share several similarities and differences from anvils reported from Bossou, Republic of Guinea. Djouroutou hammerstones possess significantly fewer discrete use-wear areas and less relative surface area affected by percussive damage compared with tools from Bossou. There is no significant difference between anvils from Djouroutou and those from Bossou in these measures (table 4). Conversely, hammerstones from Djouroutou show no significant difference in PA values compared with tools from Bossou, while these values on anvils from Djouroutou are significantly greater. Additionally, the distance of use-wear from the edge of the active surfaces for both hammerstones and anvils at Djouroutou is significantly greater compared with tools from Bossou. A principal component analysis applied to the above measures differentiates between both the hammerstones and anvils at Djouroutou as well as tools from Bossou, with PC1 and PC2 accounting for 81.85% of the variation within the sample (figure 4). PC1 is positively correlated with the number of discrete use-wear areas, and the various measures of distance to the centre and edge of active surfaces, and is negatively correlated with PA and density. PC2 is positively correlated with the number of discrete use-wear areas, PA, density and DAC, and is negatively correlated with DAE measures. The differences in these distances can be explained by the overall larger dimensions of both hammerstones and anvils at Djouroutou compared with Bossou (figure 6).

**Table 4.** Results of Mann–Whitney U tests comparing the frequency and spatial patterning of percussive damage on hammerstones and anvils from Djouroutou and anvils from Bossou (significant results = italics).

measure	Djouroutou hammerstone versus Bossou tools		Djouroutou anvil versus Bossou tools	
	U	*p*-value	U	*p*-value
number of discrete use-wear areas	106	*<0.001*	27.5	1.000
PA	120	*<0.001*	42	0.120
DAC mean	84	0.131	6	*0.009*
DAC minimum	0	*<0.001*	0	*<0.001*
DAC maximum	104	*0.003*	11	0.054
DAE mean	7	*<0.001*	0	*<0.001*
DAE minimum	0	*<0.001*	0	*<0.001*
DAE maximum	27	*0.033*	0	*0.002*

## 4. Discussion

Percussive technology, both flaking and non-flaking, has played a fundamental role in the biological and cultural evolution of hominins [6,8,18]. A primary aim of primate archaeology is to develop reference models that can be used to identify and interpret percussive artefacts in the Plio-Pleistocene archaeological record [31,32,76]. Our study contributes to this growing referential database of stone tools used by wild primates that can be used to both increase our understanding of chimpanzee material culture in a wider context as well as foster comparative studies with and between other chimpanzee groups and archaeological examples of percussive technology.

### 4.1. Percussive stone tools at Djouroutou

Our study has shown that there are clear differences in the techno-typological attributes as well as the use-wear patterning between hammerstones and anvils used by chimpanzees at Djouroutou. Previous studies have shown that chimpanzees preferentially select hammerstones of specific size and mass depending on the hardness of nut species [44]. The dimensional patterning of the Djouroutou hammerstones is consistent with this observation. Hammerstones used to crack coula nuts, the softest of the nut species [58,77], are notably smaller compared with those used to crack the harder panda and parinari nuts [77]. Hammerstones at Djouroutou generally possess one or two flat active surfaces with discrete centrally located areas of damage or discoloration due to adhering nut debris. In the majority of cases, two active surfaces are located on directly opposing sides. Hammerstones on fine-grained raw materials possess less percussive damage in the form of pitting compared with more coarse-grained raw materials such as quartzite and metamorphosed granite. Unintentional flake detachments caused by miss-hits are only present on fine-grained raw materials. This is in agreement with previous studies which note that flake removals during wild chimpanzee percussive activities are more likely to occur on homogeneous raw materials [40].

Differently from hammerstones, anvils used at Djouroutou tend to be larger unmovable embedded boulders located within close proximity to one or more nut trees [60]. These anvils possess a single flat active surface, characterized by multiple discrete pitted regions. These pits possess a generally circular morphology with an undulating base, and in some cases, multiple pits have conjoined to form more elongated or irregularly shaped larger pitted regions. These irregular-shaped pits may be associated with repeated use of the same location of the anvil surface over time.

Spatially, hammerstone and anvil percussive damage at Djouroutou differ substantially. In the majority of cases, discrete percussive damaged areas on hammerstones are located centrally on the active surface with damage spread over a wider area on anvils. Analysis of the three-dimensional surface morphology of damaged areas on both hammerstones and anvils shows that anvils possess significantly deeper and steeper pitted regions compared with hammerstones. This is potentially related to an increased use life of anvils, where established pitted regions are re-used over time, facilitated by their persistent presence on the forest floor. Experimental studies on capuchin pitted anvils have shown that individual pits develop so that their maximum depth equals the maximum dimension of the cracked nut. At this point, pits cease to get any deeper [78]. Instead, pits may become more shallow as the surrounding active surface is mechanically removed by increased instances of hammerstone impacts [78]. As such, the depth of pits on both chimpanzee hammers and anvils where crushing is prevalent around them may not always be a direct indicator of use extent. Additionally, raw material hardness and granularity would affect this process [69].

The data presented in this study indicate that there are common attributes that can be used to differentiate active and passive nut-cracking tools. All anvils in this study possessed multiple discrete areas of use-wear on their active surface. These were distributed across the active surface, and in many cases, multiple pitted areas combined to form larger irregularly shaped pits. This damage pattern can be associated with repeated reuse of the same active surfaces over time. This observation is in line with other examples of primate stone anvils, such as those used repeatedly by capuchin monkeys (*Sapajus libidinosus*) [78,79]. Hammerstones, however, normally possessed a singular confined area of damage located centrally on the active plane. Additionally, only hammerstones possess damage located on two opposed active planes, while all anvils possess damage on a singular surface. Even in cases where anvils were small enough to be used on both sides, the damage was only located on a single plane.

At Djouroutou, chimpanzees use portable stone hammers, while the majority of stone anvils are stationary. Our study indicates that in some cases hammerstones and anvils possess a more complex use life. As noted above, a major differentiating feature between hammerstones and anvils at Djouroutou is the combination of percussive damage location (both distance from anvil centre and distance from anvil edge), and the frequency and depth of discrete damaged areas (pitting). Based on these criteria, there are two examples of hammerstones presented in this study which show a combination of both hammerstone and anvil damage patterns developed during different periods in their use life. The first is a complete granodiorite hammerstone, used to crack coula nuts, which shows a clear centrally located area of macro-residue around a shallow pit associated with its use as a hammerstone. Two additional deeper off-centre pits, identified through surface topographical analysis and not associated with macro-residue, attest to this surface having been used as an anvil. The second example is a large fractured metamorphosed granite hammerstone used to crack panda nuts, which possess a singular large central pit on one active plane, with clear recent pitting and crushing of the grains. However, a cluster of five discrete off-centre pitted regions, covered by a layer of algae and moss growth, is located on the opposite plane. These results indicate that, in some cases, at Djouroutou, stone tools used as anvils are transported and re-used as hammerstones. This is in line with previous observations that functional distinctions between hammers and anvils may not be ridged for chimpanzees, with each tool type being interchangeable to a degree throughout their use life depending on their functional dimensions [44,51].

It has been shown that chimpanzees possess a durable archaeological record in their own right [52,65] primarily consisting of hammerstone fragments [40]. The large stationary or rarely moved anvils used by the Djouroutou chimpanzees to crack nuts possess substantial modification through use. The larger of these artefacts may take a considerable duration to fully enter the archaeological record and as such would persist in the landscape as identifiable activity areas, allowing for continuous use and reuse over time. Furthermore, these artefacts would probably preserve long after the death of the associated nut trees, making them a potentially invaluable marker of past chimpanzee nut-cracking behaviours in future primate archaeological endeavours at Djouroutou.

### 4.2. Percussive technology compared between chimpanzee populations

As the referential sample of chimpanzee percussive tools increases, detailed comparisons of the artefactual records between chimpanzee groups become possible. This allows a refinement of our understanding of regional differences in damage patterns on chimpanzee stone tools_._

When compared with the published stone tools from Bossou, the only other site that has undergone similar techno-typological and surface morphometric characterization [39], some notable differences and similarities are apparent. First, the range of raw materials used for tools by Bossou chimpanzees is more restricted compared with those used at Djouroutou. At Bossou, both ironstone and amphibolite are used [39], while quartzite, granodiorite and metamorphosed granite and ironstone are used at Djouroutou [60]. This difference stems from the local availability of raw materials dictated by the underlying geology of each area. Bossou percussive tools differ significantly in the frequency of use-wear areas, the relative degree of percussive damage and in distances of use-wear to the centre and edges of the active surfaces. Bossou tools and Djouroutou anvils are more similar compared with Djouroutou hammerstones in terms of the frequency and relative degree of use-wear areas. On the other hand, both Bossou tools and Djouroutou hammerstones possess more centrally located damage patterns compared with Djouroutou anvils. The tools from Bossou possess damage considerably closer to the edges of the active surfaces compared with both hammerstones and anvils from Djouroutou. The differences between the percussive tools from both Bossou and Djouroutou may not reflect behavioural differences between the groups. Instead, as the reported tools from Bossou were derived from a field laboratory [39], the degree and characterization of damage may have been influenced by the level of provisioning within the field laboratory. Furthermore, these differences may also have been affected by the raw material used at each site as well as the frequency of tool use.

Nevertheless, in their characterization of percussive tools from Bossou, Benito-Calvo *et al.* [39] note that, although there is a degree of overlap between anvils and hammers, anvils possess deeper and steeper depressions compared with hammerstones. The same differentiation is seen between the anvils and hammers from Djouroutou reported in this study. These similarities across behavioural locations, nut species and raw materials, suggest that chimpanzee anvils may share common use-wear patterns that differentiate them from hammerstones. The clear differences noted above potentially indicate a degree of regional variation. These differences may be associated with regional raw material differences and availability, differences in how both hammerstones and anvils are used and the hardness of the nuts cracked.

### 4.3. Relevance to hominin percussive technology

The results of this study can also be discussed in relation to percussive artefacts identified at various Plio-Pleistocene archaeological sites that share both similarities and differences with the hammerstones and anvils presented here.

Archaeological percussive artefacts are often classified as either active or passive elements using the overall morphology and size of the artefacts themselves [1]. Traditionally, passive elements have been defined as large, tabular or trapezoid and possess flat stabilizing bases [7,15,22]. Often these artefacts possess flake detachments around their vertical edges [7,22]. Active elements, however, are defined as being smaller and possessing rounded or irregular morphologies [1,7,15]. Damage patterns identified on archaeological anvils are diverse. Some anvils identified at Olduvai and Úbeidiya (Israel) show damage along the edges of active surfaces [7,23]. In some cases, artefacts classified as anvils possess pitted damage on two opposed surfaces. Examples of such artefacts come from the Acheulean site of Gesher Benot Ya'qov [22] where thin basalt slabs possess damage across their surfaces and on multiple active planes. These artefacts are interpreted as nut-cracking anvils [13]. Several factors may account for this variation of percussive damage on anvils, including different uses or targets [17], their reuse over time or differing mechanical actions inflicted upon them [33]. It is also understood that convex active surfaces are desirable characteristics for active hammerstones used for stone tool production (flaking) [7].

The chimpanzee hammerstones described in this study fall within the range of dimensions, percussive damage and techno-morphological criteria used to identify some archaeological anvils in the Plio-Pleistocene archaeological record. The Djouroutou hammerstones often possess a tabular morphology and retain varying degrees of damage visible on their active surface(s). Furthermore, at Djouroutou, some hammerstones possess flake detachments located around the periphery of the active surfaces. This occurs when the hammerstone strikes the anvil either due to a miss-hit or following a successful strike [33]. In many cases, these flake detachments mimic examples of anvil flake detachments in the archaeological record [7,22]. Furthermore, these hammerstones often possess damage on multiple opposed active surfaces. Based on these data, it is feasible that some percussive tools with damage on opposed planes may have been used as active hammerstones as opposed to passive anvils. The primate stone tool record, furthermore, shows that, for some percussive tasks such as nut-cracking, flat surfaces on hammerstones are preferable. As such, a flat active surface on a larger percussive stone tool should not be used as a criterion for assigning an artefact as a passive element. Such artefacts were undoubtedly manipulated in myriad ways and potentially for multiple functions [10]. By expanding our understanding of the varying characteristics of primate percussive tools, it is possible to highlight the potential variability associated with the techno-morphological and use-wear patterns of percussive artefacts in the Plio-Pleistocene archaeological record.

Finally, this study reports on a single large, fine-grained quartzite hammerstone used to crack parinari nuts which is devoid of macro percussive damage or modification. This hammerstone was found resting on top of a parinari tree root used as an anvil alongside fresh nut debris. Additionally, this particular nut-cracking site was located on a substantial granite inselberg which was devoid of nearby naturally occurring quartzite sources. When viewed geologically, this artefact does not conform to the surrounding natural sediment type or clast size, as it was transported to the site by a primate. The presence of such an artefact at a percussive behavioural location highlights a potential behavioural interpretation for the presence of seemingly unmodified stones in Plio-Pleistocene contexts [1,80–84]. Percussive behaviours can in some cases result in the accumulation of stones devoid of clear evidence of use. Future comparative studies of non- and minimally modified primate percussive stone tools and Plio-Pleistocene unmodified stones or manuports may shed further light on this matter and offer new avenues to interpret unmodified stones in Plio-Pleistocene sites.

## 5. Conclusion

We have shown that the stone tools used by the chimpanzees of Djouroutou in the Taï National Park for nut-cracking possess a range of techno-typological and percussive damage patterns that differentiate hammers from anvils. Furthermore, these percussive tools differ from those used by chimpanzees of the Bossou Forest. These differences are probably affected by the range of raw materials used as well as the different hardness of the nut species cracked. Furthermore, our analysis shows that the Djouroutou chimpanzees occasionally re-purpose anvils as hammerstones, attesting to the long use-life histories of chimpanzee stone tools. Additionally, most anvils in Djouroutou would remain in the chimpanzee archaeological record long after a nut-cracking site is abandoned, making them durable signatures of past behaviours. From an archaeological perspective, the chimpanzee hammerstones from Djouroutou share several similarities and differences with known Plio-Pleistocene examples of percussive artefacts and contribute to increasing our referential framework of variation within percussive technology. This, in turn, can be used to better understand the range of possible percussive behaviours undertaken by our Plio-Pleistocene ancestors.

## Data Availability

The datasets supporting this article have been uploaded as part of the electronic supplementary material [85]. These data are deposited at Dryad: https://doi.org/10.5061/dryad.x3ffbg7nc [86].
